# Somatosensation in OA: exploring the relationships of pain sensitization, vibratory perception and spontaneous pain

**DOI:** 10.1186/s12891-018-2206-4

**Published:** 2018-08-25

**Authors:** Anisha B. Dua, Tuhina Neogi, Rachel A. Mikolaitis, Joel A. Block, Najia Shakoor

**Affiliations:** 1 Section of Rheumatology, University of Chicago, 5841 S Maryland Ave, MC0930, Chicago, IL 60637 USA; 2 Section of Clinical Epidemiology Research and Training Unit, Boston University School of Medicine, Boston, MA USA; 3 InventIv Health, Clinical, Abbvie, Chicago, IL USA; 4 Division of Rheumatology, Rush Medical College, Chicago, IL USA

**Keywords:** Osteoarthritis, Pain, Somatosensory measures, Allodynia

## Abstract

**Background:**

Pain in osteoarthritis (OA) remains poorly understood. Different types of somatosensory alterations exist in OA including hyperesthesia and increased sensitivity to painful stimuli as well as those of decreased sensitivity to cutaneous stimuli including vibratory perception threshold. The relationship between these different somatosensory measures has not been previously evaluated in OA. In this observational study, we evaluated relationships between vibratory perception (VPT), pressure pain detection thresholds (PPT), allodynia and subjective pain in knee OA.

**Methods:**

Forty-two persons with moderate to severe knee OA and 12 controls without OA were evaluated. VPT was measured using a biothesiometer. Allodynia was measured by application of a 60 g Von Frey monofilament repeatedly to predetermined sites. PPTs were measured using a pressure algometer.

**Results:**

Increased vibratory acuity was associated with lower PPTs and presence of allodynia. Allodynia was more common in OA than controls (54.8% vs 16.6%, *p* = 0.024 in the ipsilateral knee, and 42.9% vs 0%, *p* = 0.005 in the contralateral knee). OA participants with allodynia had lower PPTs than those without allodynia. In those with OA, spontaneous knee pain was associated with lower PPTs and with allodynia.

**Conclusion:**

This study confirms the presence of somatosensory alterations in OA. Sensory alterations (vibratory perception) were shown to be related to nociceptive alterations (sensitization) in OA, showing a general increased sensitivity to cutaneous mechanical stimulation. Understanding these relationships is an important step in delineating the complicated pathophysiology of pain processing in OA.

## Background

Osteoarthritis (OA) is the most common chronic arthropathy worldwide, and pain is the most disabling symptom [1]. Mechanisms of pain in OA are poorly understood, and there is only a weak association between pain and radiographic knee OA [2]. A better understanding of somatosensory pathways and their roles in OA and OA-related pain may help improve our understanding of OA pathogenesis and our management of OA.

“Somatosensory” has a broad application when referring to alterations of the nervous system that have been shown to be present in clinical OA. First, “somatosensory alterations” could refer to those of specific pain processing pathways. Studies have suggested that abnormal excitability in the pain pathways of the peripheral and central nervous system play an important role in OA pain [3–5]. Continuous nociceptive input in OA could affect neuropeptide release from nerve endings, neuroplasticity, increases in synaptic strength and lowered firing thresholds of the dorsal horns. This could lead to changes in pain thresholds, and spreading of pain to uninjured sites [6]. The continuous input in chronic painful states such as OA can lead to heightened responsiveness, or “sensitization”, of peripheral nociceptors (i.e., peripheral sensitization) and nociceptors in the central nervous system resulting in hypersensitivity to stimuli, responsiveness to non-noxious stimuli, and increased pain response evoked by stimuli outside the area of injury (i.e., central sensitization) [7–9]. Sensitization may be expressed clinically as allodynia (painful response resulting from a normally innocuous stimulus) and hyperalgesia (enhanced pain response to a noxious stimulus). Previous studies of symptomatic knee OA have demonstrated findings suggestive of sensitization or heightened pain sensitivity, including lower pressure pain thresholds (PPT) and the presence of mechanical allodynia [8, 10–12].

In addition to the nociceptive sensory alterations in OA, there is extensive literature of other types of “somatosensory” alterations that exist in OA, including deficits in such functions as vibratory perception and proprioception [13, 14]. We have previously shown that subjects with knee OA and hip OA have generalized vibratory sense deficits with impaired vibratory perception threshold (VPT) at both the upper and lower extremities compared to age-matched control subjects [14, 15].

Interestingly, the paradox between the different types of somatosensory alterations in OA is the existence of both “increased sensitivity” to some cutaneous stimuli (pressure and pain) while there is “decreased” sensory function in other measures such as sensation of vibration and proprioceptive acuity. Some studies in neuropathic pain have previously demonstrated the coexistence of somatosensory “profiles” with both loss and gain of sensation or sensitivity [16]. The relationships between pain sensitization and vibratory sense alterations in OA are not clear and have not been previously evaluated. Although vibratory sense may have a mechanical role similar to the hypothesized role of proprioceptive deficits in OA, alterations in vibratory sense may also play a role in pain processing in OA.

Here we explore the relationship between nociceptive alterations and other somatosensory alterations, specifically, vibratory sense in OA. We evaluate vibration sense, allodynia, pressure pain and subjective pain in knee OA participants and in healthy, pain-free controls. Our primary hypothesis is that vibratory acuity will be inversely associated with other somatosensory measures that may be reflective of sensitization. Specifically, we hypothesized that those with greater vibratory deficits will also have lower PPTs and evidence of allodynia. Our secondary hypothesis is that sensitization as well as worse vibratory acuity, indicative of chronic pain and severe OA [17–19], respectively, will be associated with higher subjective pain measures in knee OA.

## Methods

### Study sample

This study was approved through the institution’s review board for studies involving human subjects and written informed consent was obtained from all subjects. Study subjects were recruited from a single large urban academic medical center in the United States between 2011 and 2013. Subjects were recruited through referral from the Rheumatology clinic and the department’s clinical studies center. None of the subjects declined participation in the study. Study subjects were those with *symptomatic* OA of the knee, which was defined by the American College of Rheumatology’s Clinical Criteria for Classification and Reporting of OA of the knee [20] and by the presence of at least 20 mm of pain (on a 100 mm scale) while walking (corresponding to question 1 of the visual analog format of the knee-directed Western Ontario and McMaster Universities Arthritis Index (WOMAC)) [21]). In subjects with bilateral knee OA, the most symptomatic side was considered the “affected” side. Radiographic OA of the index knee was documented by anterior-posterior standing radiographs of the knees in full extension, of grade greater than or equal to 2 as defined by the Kellgren-Lawrence (KL) grading scale [22, 23]. All KL evaluations and assessments were performed by a single trained investigator (NS). Knee OA participants were excluded if they demonstrated greater than 20 mm (of 100 mm) pain while walking (corresponding to question 1 of the VAS format of the site-directed WOMAC) at any other joint besides the knees. Other exclusion criteria included the presence of an inflammatory arthropathy or neuropathy, history of any lower extremity joint replacement or diabetes mellitus.

Controls were recruited from Rheumatology clinic as well as personal referrals from clinic staff age-matched control participants were included in the study if they had pain less than 20 mm (of 100 mm) at the knee, hip and ankle while walking (corresponding to question 1 of the VAS format of the site-specific WOMAC) and did not have radiographic knee OA, documented by KL grade of 0 or 1 on knee radiograph. The index knee in the control group was the right knee and the contralateral knee was the left knee. They had the same exclusion criteria as the OA participants.

### Mechanical allodynia to repetitive stimulation

Allodynia was assessed using a 60 g Von Frey monofilament, which is an innocuous mechanical stimulus in healthy individuals. The sites evaluated were the tibial tuberosities and the right radial styloid. The Von Frey monofilament was applied perpendicularly to the skin with enough pressure to make it bend. The monofilament was then applied repeatedly to the same site at a rate of 1 Hz for 30 s. Subjects provided a numerical pain rating at the end of this train of stimulations. Allodynia was considered to be present when participants answered “yes” to the question “do you consider this painful?” Participants provided a numerical pain rating at the end of this train of stimulations, rating the extent of their pain on a scale of 0 to10 after each trial (“0” representing no pain). The procedure was repeated twice at each site and the mean of the pain rating values was used for analysis. The intraclass correlation coefficient (ICC) for this method at our center was 0.49–0.66 between initial and repeat testing on separate days.

### Pressure pain detection thresholds

Pressure pain detection thresholds (PPT) were defined by applying a pressure algometer (FPIX, Wagner Instruments, Greenwich, CT; 1cm^2^ hard rubber tip) on each anatomic site at a rate of 0.5 kg/second as the point at which the subject reported the pressure first changed to pain [24]. The sites tested were the medial joint line and tibial tuberosity of each knee and both radial styloids. The PPT in kilograms of force (kg/force) was obtained three times at each site. The mean value of the three trials was used in analysis. The ICC for PPT evaluation at our center was 0.70–0.96 between initial and repeat testing on separate days.

### Subjective pain assessments

All participants completed questionnaires regarding knee pain and function, including the question “Do you feel spontaneous pain in your knee?” Subjects completed the WOMAC visual analog scale for evaluation of pain at both knees, both ankles and both hips. The WOMAC is a standardized and validated questionnaire for assessment of pain and function in lower extremity OA [21, 25, 26] and site-specific adaptation of the WOMAC has proven useful and feasible [27, 28].

### Vibration perception

VPT was measured using a biothesiometer (Bio-Medical Instrument Co., Newberry, Ohio) operating at a frequency of 120 Hz according to previously published methods [14]. The following anatomic sites were tested: the first metatarsophalangeal joint, medial malleolus, lateral malleolus, medial femoral condyle, tibial tuberosity, and lateral femoral condyle bilaterally. The bilateral radial styloids were used as control sites. The applicator tip of the machine was placed at the predetermined anatomic site. The biothesiometer voltage was set at “0” volts and the voltage output was increased by 1 V/second until subjects verbally reported their first sensation of vibration. Each site was tested twice and the mean value was recorded and used for analysis. Higher voltage represented worse vibratory sense acuity. The ICC was high, 0.96–0.99, between initial and repeat testing on separate days.

All qualitative sensory testing (QST) assessments were performed by one of two trained investigator (AD, RM), and subjects were given standardized instructions prior to testing.

### Statistical analyses

Power calculations were based upon the difference in means in previous studies available in 2011 at initiation of the study that had evaluated allodynia and PPT’s in OA subjects [10, 12]. Using conservative estimates, we based our power calculation on a mean PPT (kg/cm^2^) in the control group of 5 and the OA group of 3.8 with a combined standard deviation of 1.3. Inclusion of 37 participants with OA and 10 age-matched healthy controls was estimated to provide 80% power to detect differences between the groups (α = 0.05 and sampling ratio of approximately 3:1 OA to control participants). There are no prior studies evaluating the correlations between VPT, PPTs, and allodynia in OA participants. Therefore, estimating a small to moderate effect size (0.15), power of 0.80 and α = 0.05, 40 participants with OA would be needed to examine these relationships.

Independent samples t-test and Chi-square tests were used to compare the OA with the control group. Pearson and Spearman correlations were used to evaluate bivariate associations within the OA group. *P* < 0.05 was considered to be statistically significant. All values are reported as mean ± standard deviation.

## Results

Forty-two OA participants (mean age 54 ± 8 years, 13 males/29 females) and twelve controls (mean age 53 ± 11 years, 3 males/9 females) were studied. A majority of the participants had bilateral knee OA. The KL grade at the index knee included 26 KL 2 and 16 KL 3 knees and at the contralateral knee, 2 KL 1, 28 KL 2, and 12 KL 3. Mean WOMAC pain score at the index knee (0 to 100 mm ± SD) was 183 ± 110 mm and at the contralateral knee was 129 ± 111 mm. WOMAC pain at the ankles and hips was less than 60 mm out of 500 mm in the OA group. The control participants had no knee pain on full WOMAC evaluation (mean < 1 mm out of 500 mm at both knees).

Significantly more OA participants demonstrated mechanical allodynia (responded yes to the question “do you consider this painful?” after completion of the Von Frey filament stimulus, (*n* = 23) compared with controls at the ipsilateral knee (54.8% vs 16.6%, *p* = 0.024) and contralateral knee (42.9% vs 0%, *p* = 0.005). Pressure pain thresholds (PPT) were lower in the OA group but did not reach statistical significance (affected tibial tuberosity 3.81 ± 1.63 vs 4.62 ± 1.37 kg/force, *p* = 0.09 and affected medial joint line 2.73 ± 1.55 vs 3.65 ± 1.57 kg/force, *p* = 0.09).

VPT and PPT were directly correlated at several anatomic sites (Table 1), in that those with increased sensitivity to vibration also had lower pressure pain thresholds. Similarly, VPT was lower at the first metatarsophalangeal joint (MTP) in those who had allodynia at the ipsilateral tibial tuberosity compared with those that did not (8.2 ± 3.3 vs 12.1 ± 4.8 V, *p* = 0.005).

**Table 1:** Correlation between VPT at Multiple Sites with PPT at the Ispilateral Tibial Tuberosity and Medial Joint Line in OA participants*

	PPT Ipsilateral tibial tuberosity Spearman’s rho (*p* value)	PPT Ipsilateral medial joint line Spearman’s rho (*p* value)
VPT Ipsilateral MTP	0.272 (0.081)	0.310 (0.046)
VPT Ipsilateral medial ankle	0.416 (0.006)	0.476 (0.001)
VPT Ipsilateral lateral ankle	0.210 (0.182)	0.193 (0.221)
VPT Ipsilateral medial knee	0.338(0.030)	0.389 (0.010)
VPT Ipsilateral lateral knee	0.432 (0.004)	0.405 (0.008)
VPT Ipsilateral tibial tuberosity	0.350 (0.023)	0.268 (0.086)

*Association between increased sensitivity to vibration and lower pressure detection thresholds

OA participants with allodynia at the ipsilateral tibial tuberosity (*n* = 23) had higher WOMAC pain scores at the affected knee compared to OA participants without allodynia (217 ± 111 vs 142 ± 97 mm, *p* = 0.025). This relationship was not seen at the other anatomic sites.

Bivariate correlations showed no relationship between WOMAC pain score at the ipsilateral knee in OA participants and PPT (rho = − 0.115 to − 0.139, *p* > 0.05) or VPT (rho = − 0.051 to − 0.219, *p* > 0.05) at any of the several sites tested.

Further exploratory analyses were performed. OA participants with allodynia at the ipsilateral tibial tuberosity had significantly lower PPTs at multiple sites compared with those without allodynia (Table 2). Information regarding spontaneous pain was also evaluated and was available in 35 of the 42 OA participants due to the questionnaire being added later in the study. It was available on all control participants. The experience of spontaneous pain in the knee was only observed in OA participants (74.3% in OA vs 0% in controls, *p* = 0.001). In the OA group, participants with spontaneous knee pain had significantly lower PPTs than those without spontaneous knee pain at all ipsilateral and contralateral sites (Table 3). Spontaneous knee pain was also associated with presence of allodynia as well as the pain rating post-stimulation at the ipsilateral and contralateral tibial tuberosities (Table 3). An association between VPT with spontaneous pain was not observed.

**Table 2:** Relationship between Allodynia and Pain Pressure Threshold in OA participants

Pressure Pain Threshold (kilograms of force), (Mean ± SD)	Presence of Allodynia at Ipsilateral tibial tuberosity		*P*-value
	Yes (n = 23)	No (*n* = 19)	
Ispilateral radial styloid	2.56 ± 1.19	3.71 ± 1.53	*0.01*
Contralateral radial styloid	2.51 ± 1.26	3.75 ± 1.50	*0.007*
Ispilateral tibial tuberosity	3.16 ± 1.56	4.59 ± 1.39	*0.003*
Contralateral tibial tuberosity	3.57 ± 1.61	4.48 ± 1.32	0.052
Ispilateral medial joint line	2.16 ± 1.23	3.44 ± 1.64	*0.008*
Contralateral medial joint Line	2.54 ± 1.46	3.77 ± 11.81	*0.02*

*significant relationship between allodynia at the ipsilateral tibial tuberosity and lower PPT at tested site

**Table 3:** Relationship Between Spontaneous pain and Pressure Pain Thresholds and Allodynia in OA participants

	Spontaneous Pain		*P* value
	Yes (*n* = 26)	No (*n* = 9)	
Pressure Pain Threshold, Radial Styloid (kgf)*, (Mean ± SD)	2.42 ± 1.07	4.39 ± .1.25	0.001
Pressure Pain Threshold, Medial Joint Line (kgf), (Mean ± SD)	2.05 ± 1.08	3.98 ± 1.61	0.001
Pressure Pain Threshold Tibial Tuberosity (kgf), (Mean ± SD)	3.52 ± 1.44	5.01 ± 1.11	0.005
Presence of Allodynia, Ipsilateral Tibial Tuberosity	69.20%	30.70%	0.012
Pain Rating, Ipsilateral Tibial Tuberosity Scale after Repeated Mechanical Stimulus (Mean ± SD)	4.3 ± 2.6	1.6 ± 1.7	0.003
Presence of Allodynia, Contralateral Tibial Tuberosity	65.40%	11.10%	0.007
Pain Rating, Contalateral Tibial Tuberosity Scale after Repeated Mechanical Stimulus (Mean ± SD)	3.7 ± 2.3	1.4 ± 1.3	0.001

*kgf kilogram force

## Discussion

Understanding relationships between various somatosensory measures as well as subjective pain in knee OA can provide insights into pain processing in OA [10]. Central and peripheral sensitization, which are due to alterations in central and peripheral pain processing resulting in allodynia and hyperalgesia [6], have been increasingly recognized as a potential contributor to the experience of pain in those with knee OA.

We hypothesized that in OA participants, poor vibratory sense would be associated with higher pain sensitivity (lower pain threshold) and allodynia. Instead, our study demonstrated that *increased* vibratory acuity, or increased sensitivity to vibration, was associated with increased pain sensitivity (lower pain detection thresholds) as well as the presence of allodynia to repetitive stimulation.

Poor VPT [14], lower PPT and hyperalgesia have been reported in OA [10–12]. The association between these different somatosensory measures is likely complicated as has been suggested by previous studies in chronic neuropathies in which combinations of heightened responses to one somatosensory measure coexisted with dampened responses to a different measure [16]. This is initially what we hypothesized to be the case in OA. However, in this study, the direction of alterations appeared to parallel one another, such that when vibration was felt more easily (higher acuity), so was pain (lower PPTs) as well as the experience of pain with a usually non-noxious stimulus (allodynia). This suggests that the OA participants may have had widespread sensitization to a variety of types of mechanical stimulation. This was a surprising observation in light of our previous findings that subjects with painful symptomatic knee OA have decreased sensitivity to vibration compared with age-matched controls [14]. Thus far, vibratory deficits in OA have been hypothesized to play a mechanical role in OA with suggestion that alterations in sensory input would lead to aberrant mechanics and possible OA progression [18]. However, vibratory acuity may have a different nociceptive role or association in OA and may be involved in or be affected by central pain processing in OA, as suggested by this study’s results. In larger groups, it would be important to examine whether these relationships between vibration and other somatosensory or sensitization measures vary depending on the severity or stage of OA. Notably, the OA participants in this study had primary knee OA without much pain at other lower extremity joints. Populations with multi-articular lower extremity OA or chronic widespread pain may differ on these associations.

In our cohort, OA participants with allodynia at the ipsilateral knee had significantly lower pressure pain thresholds at all ipsilateral and contralateral sites, suggesting that there is a relationship between these different measures of sensitization/pain sensitivity. Further, those with OA and spontaneous pain had significantly lower pressure pain thresholds at ipsilateral and contralateral sites, greater likelihood of allodynia and higher pain ratings with repeated mechanical stimulation than those without spontaneous pain. These relationships suggest that the presence of spontaneous pain in knee OA correlates with measures thought to reflect sensitization. With repeated nocicecptive stimulation, high threshold polymodal C fibers may undergo changes that result in enhanced sensitivity, lowered thresholds for activation, and prolonged and enhanced response to the stimulation [29]. These neuroplastic changes may explain why OA participants experience heightened pain sensitivity and spontaneous pain. In this study, traditional measures of self-reported knee OA pain, the WOMAC pain scale, did not correlate as consistently with the somatosensory measures tested. This is in contrast to some prior studies [10, 12, 30]. In particular, in a large systematic review, Fingleton et al. demonstrated there was greater pressure pain sensitivity in a high symptom severity group compared to a low symptomatic group [8]. In our study, there was some association noted between allodynia and WOMAC pain and some of the relationships between PPTs and WOMAC pain approached significance. It could be that with greater numbers of participants, these relationships would be more evident, and small size is a limitation of our study. Nevertheless, the strong association observed between spontaneous pain, PPT, and allodynia suggests that perhaps more descriptive characteristics and temporal characteristics of pain should be examined as markers of sensitization. Certainly these findings provide further support for a role for quantitative sensory assessments of pain as being distinct and complementary to subjective reports of pain ratings.

Our study has several limitations. We did not control for all potential confounders or comorbidities such as specific nervous system disorders or baseline medications, though we did exclude those subjects who had inflammatory arthritis, neuropathy, diabetes, or any lower extremity joint replacement. We did not have information on duration of disease. Some of our results are based on subgroup analyses that were not originally part of our original power calculations. We looked at one measure of sensory function, vibratory sense, and future studies may want to investigate other measures such as balance and proprioception. Although the question regarding “spontaneous pain” in our study is not necessarily a validated questionnaire tool, and it may be subject to variations in interpretation [31], it nonetheless appeared to be attested to frequently in the OA participants and appeared to separate them from controls. Future studies should look at additional ways to help detail characteristics of subjective pain in OA and how pain characteristics may be markers of central or peripheral sensitization.

## Conclusions

In summary, vibratory acuity in knee OA appears to be associated with the presence of sensitization and pain sensitivity in OA, such that there is a generalized increase in sensitivity to cutaneous mechanical stimulation, including vibratory sense. This relationship between nociceptive sensory alterations and other somatosensory alterations in OA had not previously been evaluated. The presence of spontaneous pain may be an indicator of the presence of sensitization in OA. An understanding of the relationships between spontaneous pain, stimulus-evoked pain, and somatosensory measures will be critical to fully understand pain processing in OA.

## Abbreviations:

KL: Kellgren-Lawrence
MTP: Metatarsophalangeal joint
OA: Osteoarthritis
PPT: Pain pressure thresholds
VPT: Vibratory perception threshold
WOMAC: Western Ontario and McMaster Universities Arthritis Index
